# Medical Items

**Published:** 1917-04

**Authors:** 


### MEDICAL ITEMS

### AN IMPORTANT HYPNOTIC

That often intractable symptom of nervous disorder, insomnia, suggests the use of Chloretone in preference to hypnotics of the coal-tar series, for the reason that the former is not depressant to the heart and respiration and is not toxic in ordinary therapeutic doses. It produces peaceful slumber closely resembling the natural process, the patient awakening refreshed and rejuvenated. It does not disturb the indigestion, produces no objectionable after-effects, and does not cause habit formation.

Administered internally, Chloretone passes unchanged into the circulation and is deposited in considerable quantities in the cerebral tissue, the results being that the patient falls into a profound sleep. It appears to be decomposed with the body, for, volatile as it is, we do not find it in the expired air nor can it be recognized in the urine.

Chloretone is useful in the treatment of the insomnia of acute mania, periodic mania, senile dementia, the motor excitement of general paresis, and alcoholism. It is especially beneficial in controlling sleeplessness due to pain—as in cancer, tabes dorsalis and neuralgias—and to mental strain or worry.

As doubtless most physicians know, Chloreton is a product of the laboratories of Parke, Davis & Co. It is supplied in crystal form—in ounce vials and in capsules of three grains and five grains· Because of its camphoraceous flavor it is perhaps best to administer it in capsules.

---

### MODERN TREATMENT OF WHOOPING COUGH

Up to 1916 there was a steady decrease in the number of deaths resulting from measles, scarlet fever and diphtheria, notwithstanding the increase in population, while the mortality from whooping cough had increased. This fact is all the more pertinent when we realize in the 17th century Willis, describing ''chin cough'' as the disease was then called, says that the mortality was gradually increasing due to the fact that the treatment was drifting into the hands of quacks and old women largely because o fwant of successful treatment by the practicing physician.

Two hundred and thirty-eight years later, in the twentieth century, a remedy for whooping cough appeared in the British Medical Journal, over the signature of a physician. He recommended the wearing of garlic cloves in the stockings· The efficacy of the treatment is further impressed upon one by the statement that within a half hour after the garlic has been placed in the stockings, a marked odor of garlic could be detected, on the breath.

Sufficient evidence has now been collected to demonstrate the value of the modern tratment of whooping cough by means of inoculations of pertussis vaccine for prophylaxis against and immunization in whooping cough.

### BROMIDIA AND EXTEMPORANEOUSLY PREPARED MIXTURES.

The advantage offered by Bromidia (Battle) over extemporaneously prepared mixtures of the bromides lies in the fact of purity of the bromide content, greater and more accurate care in compounding the product, and the scientific fortifying of the bromide content with other sypergistic agents. For these reasons. Bromidia (Battle) is not only more acceptable to the average patient than an extemporaneously prepared bromide mixture, but, further still, much more potent in effect. These points of superiority make it possible to crowd Bromidia (Battle) in dosage and to continue it for long periods without the usually experienced untoward effect of the more crudely prepared bromide mixtures.

With the demonstration of the Bordet-Congou bacillus as the causative organism in pertussis, attention was immediately directed toward specific medication; and the results of vaccine theraphy as reported by the New York Whooping Cough Clinic, under the direction of Drs. Williams and Luttinger, prove conclusively that at last the physician has a rational effective prophylactic and therapeutic agent at his command.

Eli Lilly & Company recommend as prophylactic doses of its pertussis vaccine: First, 500 million, followed at successive three-day intervals with 1000 and 2000 million respectively. When the vaccine is used early for curative purposes and in sufficiently large doses, the paroxysms are diminished in intensity and number, improvement being manifested very rapidly after the first and second doses. The duration of the disease is shortened when vaccines are used and a greater percentage of recoveries without complications are recorded.

It is claimed by Eli Lilly & Company that the use of Lilly Pertussis Bacterial Vaccine as early as possible in epidemics will prove of great assistance in reducing morbidity and mortality and preventing the spread of the disease.

A convenient Vest Pocket Manual on Biological Therapy will be sent by Eli Lilly & Company to our readers on request.

### THE MAKING OF AMPOULES

An illuminating article on the manufacture of glaseptic ampoules of sterilized solutions, as conducted in the laboratories of Parke, Davis & Co., appears in a recent issue of Therapeutic Notes. It is noteworthy because of the emphasis placed upon the careful methods which are essential in the production of both solution and container.

"First of all," says the Notes, "the greatest care is taken in the selection of the glass from which the ampoules are made. It is of the first quality, and must be free from alkali in order to obviate any possibility of contamination or chemical action on the solution. This is vital, for it is imperative that the purity and stability of the contents of the ampoules be assured.

"The medicaments used in preparing solutions are treated with the most suitable solvents—e. g., oils, distilled water, or physiologic salt solution—and the solutions are invariably adjusted to a fixed standard of strength; that is, each contains a specific amount of medicament to a given volume, thus insuring accuracy of dose. The solutions are subjected to the process of sterilization, either by heat applied in an autoclave, at intervals, for four or five days, or by passage through a Berkefeld or Pasteur procelain filter. They are then passed into sterilized bottles, and samples are submitted to the biological department for a series of sterility tests that extend over a period of five days.

"The ampoule containers, cleansed and sterilized, are filled with the sterilized and tested solutions by machinery. The neck of each ampoule is hermetically sealed in a gas flame, and ampoules and contents are again subjected to the sterilization process, this time by the careful application of heat, care being taken to adjust the temperature of the apparatus to such a degree that the medicament will not suffer injury. The hermetically sealed container effectually protects the solution from bacterial contamination and oxidation, while the actinic effect of light is prevented by enclosure of each ampoule in an impervious cardboard carton."

As indicative of the trend in hypodermatic medication it may be noted that more than sixty sterilized solutions are now supplied by Parke, Davis & Co. in glaseptic ampoules. Convenience, asepsis, stability, accuracy of dose—solutions in ampoules appeal to modern practitioners on these grounds.

---

### THE WIDER USE OF THE BROMIDES

The great utility of the bromides when intelligently used, is not half appreciated by the average practitioner. To be sure, medical men do employ the bromides for many conditions, but to nowhere near the extent that they could· It is a mistaken idea that bromides are serviceable only for the treatment of nervous diseases. It is true, their effects may be accomplished primarily by their action on the nervous system, but when we stop to think of the essential part played by nervous factors in the maintenance of vaso-motor equilibrium, secretory activity, nutrition and so on, the service that the bromides can be called on to render in a wide variety of abnormal conditions can readily be seen.

Obviously, too great care cannot be exercised in selecting the bromide salts to be used, particularly in respect to their purity and quality. Probably one of the main reasons why the bromides have not been more generally employed is the indifferent quality and impurities which have characterized so large a portion of the available bromide preparations.

Those who have used Peacock's Bromides, however, have been able to enjoy the full advantages of this class of drugs. Made from the purest and highest grade of salts, and combined with every care and skill, Peacock's Bromides have assured the highest therapeutic efficiency with gratifying freedom from all objectionable or unpleasant effect· As a consequence, the range of use of this reliable bromide preparation has been surprising, even to those most familiar with the possibilities of bromide treatment. Many affections characterized by congestion, or having their origin in nervous irritation and the resulting spasmodic conditions, have responded to Peacock's Bromides when other measures have proven useless. So with spasmodic disorders of the intestines; many conditions that have seemed organic in character and doomed to operation, have been promptly corrected by liberal doses of Peacock's Bromides.

Many other instances showing the utility of this remedy could be cited, but lack of space forbids. Suffice it to say that there is hardly any remedy at the physician's command with a broader field of usefulness· To be able to use Peacock's Bromides in adequate dosage and for requisite periods of time, with complete absence of deleterious effect has undoubtedly been more or less responsible for the foregoing, but the fundamental fact is that the bromides in the form of Peacock's Bromides have a much more extensive field of successful applicaiton than is realized.

---

**RATIONAL TREATMENT OF BOWEL INERTIA**

There is probably no one class of drugs that has been subject to such great abuse as the laxatives. So routinely and promiscuously have remedies to increase bowel activity been prescribed that the American people have been dubbed a ''nation of physic devotees.''

It is indeed unfortunate that cathartics and laxatives have been thus employed with so little ''rhyme or reason.'' The harm that has been done is only too apparent in the countless sufferers who never have a natural bowel evacuation.

Happily a good many physicians are awakening to the desirability of directing their treatment of bowel inertia and torpidity toward rational stimulation of the physiologic functions of the intestinal tract. The results obtained point so conclusively to the wisdom of this line of treatment that the older measures which depend for their effects on irritation of the intestinal mucosa or nervous mechanism of the bowel, or both, are rapidly becoming obsolete.

Among the remedies that act by correcting insufficiency of the physiologic processes of the bowels, Prunoids undoubtedly enjoy greatest popularity. The action of this true activator of intestinal fuunctions is prompt and decided, but what is especially noteworthy, this action is without griping or any other disagreeable effects. Reactionary constipation does not follow, and unlike so many other laxative measures, Prunoids do not require continuous use in constantly increasing dosage. In fact, owing to the effect of Prunoids on the fecal mass, whereby shrinkage in size and loss of moisture are prevented, the physical conditions which promote and favor intestinal peristalsis are promptly restored. As a consequence, the functional activity of the bowels produced by Prunoids shows remarkable persistence, and a dose on two or three consecutive nights is often followed by evacuations of a most satisfying character, for several days.

It is this tendency of Prunoids—not only to produce one or more movements following each dose, but to promote physiologic regularity of the bowels—that makes this remedy so much superior to "salts," or the laxative measures commonly employed· In simple words, the use of Prunoids means the rational treatment of bowel inertia—the activation of physiologic functions.

### SLEEP WITHOUT BAD AFTER-EFFECTS.

The advantage of Pasadyne (Daniel) as a sleep-producing agent does not lie solely in its therapeutic power, but also in the fact that no bad effects follow its use. Thus, in marked insomnia Pasadyne (Daniel) may be given in full dosage and repeated without fear that disagreeable results will ensue.

Pasadyne (Daniel) possesses in high degree the power to sedate the higher centres, a feature that makes it of the utmost value in all conditions marked by sleeplessness or nervous excitability. And the prescriber may feel sure that no habit will be caused by its administration, even when continued over long periods.

A sample bottle of Pasadyne (Daniel) may be had by addressing the laboratory of John H. Daniel, Inc., Atlanta, Ga.

